# Effect of palm-based tocotrienols and tocopherol mixture supplementation on platelet aggregation in subjects with metabolic syndrome: a randomised controlled trial

**DOI:** 10.1038/s41598-017-11813-w

**Published:** 2017-09-14

**Authors:** Yee-Lin Gan, Ju-Yen Fu, Oi-Ming Lai, Boon-How Chew, Kah-Hay Yuen, Kim-Tiu Teng, Kalanithi Nesaretnam, Kanga Rani Selvaduray, Puvaneswari Meganathan

**Affiliations:** 1 Department of Bioprocess Technology, Faculty of Biotechnology and Biomolecular Sciences, Universiti Putra Malaysia, Selangor, Malaysia; 2 Nutrition Unit, Product Development and Advisory Services Division, Malaysian Palm Oil Board, 6, Persiaran Institusi, Bandar Baru Bangi, Kajang, Selangor Malaysia; 3 Institute of Bioscience, Universiti Putra Malaysia, Selangor, Malaysia; 4 Department of Family Medicine, Faculty of Medicine and Health Sciences, Universiti Putra Malaysia, Selangor, Malaysia; 5 School of Pharmaceuticals Sciences, Universiti Sains Malaysia, Pulau Pinang, Malaysia

## Abstract

Tocotrienols, the unsaturated form of vitamin E, were reported to modulate platelet aggregation and thrombotic mechanisms in pre-clinical studies. Using a Food and Drug Administration (FDA)-approved cartridge-based measurement system, a randomised, double-blind, crossover and placebo-controlled trial involving 32 metabolic syndrome adults was conducted to investigate the effect of palm-based tocotrienols and tocopherol (PTT) mixture supplementation on platelet aggregation reactivity. The participants were supplemented with 200 mg (69% tocotrienols and 31% α-tocopherol) twice daily of PTT mixture or placebo capsules for 14 days in a random order. After 14 days, each intervention was accompanied by a postprandial study, in which participants consumed 200 mg PTT mixture or placebo capsule after a meal. Blood samples were collected on day 0, day 14 and during postprandial for the measurement of platelet aggregation reactivity. Subjects went through a 15-day washout period before commencement of subsequent intervention. Fasting platelet aggregation reactivity stimulated with adenosine diphosphate (ADP) did not show substantial changes after supplementation with PTT mixture compared to placebo (p = 0.393). Concomitantly, changes in postprandial platelet aggregation reactivity remained similar between PTT mixture and placebo interventions (p = 0.408). The results of this study highlight the lack of inhibitory effect on platelet aggregation after short-term supplementation of PTT mixture in participants with metabolic syndrome.

## Introduction

Metabolic syndrome is associated with disrupted haemostasis balance indicated by higher platelet reactivity and hypercoagubility^1–3^. Changes in the regulation of thrombosis promote the development of cardiovascular diseases in individuals with metabolic syndrome, which has been classified as a real disease entity by Center for Disease Control^4–6^. Being a global public health concern, there are no clinical recommendations thus far to initiate antithrombotic therapy for individuals with metabolic syndrome. This advocates the need for investigations on supplements or nutraceuticals that have the potential to reduce the risk of cardiovascular diseases as preventive measures.

Platelet aggregation, a pathophysiologic process by which platelets adhere at the disrupted sites of vascular upon stimulation is critical for haemostatic plug formation^7^. It is well established that high platelet aggregation activity promotes formation of thrombus and disturbance of blood flow, leading to cardiovascular diseases^7, 8^. Tocotrienols, the unsaturated form of vitamin E, are principally found in several edible vegetable oils such as palm oil, rice bran oil and barley oil^9, 10^. There is documented evidence that tocotrienols exhibit antioxidant, neuroprotection, anticancer and antidiabetic attributes, which are beneficial to human’s health^10, 11^. In addition, both pre-clinical and clinical studies suggested possible modulation of mechanisms in platelet aggregation with tocotrienols^12–18^.

The inhibitory effect of tocotrienols in platelet aggregation was first reported by Qureshi *et al*.^12^, who showed that supplementation of tocotrienols-enriched diet reduced plasma thromboxane B_2_ in swine model. This finding was further corroborated by several animal models fed with tocotrienols-enriched diet^13, 14^. In a study using canine model, tocotrienols were found to cause marked reduction in ADP-stimulated platelet aggregation^15^. In hypercholesterolemic subjects, supplementation of tocotrienols significantly reduced plasma thromboxane B_2_ and platelet aggregation in response to ADP stimulation^16–18^. Despite evidence showing significant effect of tocotrienols in the modulation of platelet aggregation, several studies reported otherwise. Koba *et al*.^19^ and Watkins *et al*.^20^ showed that tocotrienols did not alter ADP-stimulated platelet aggregation in rat models. In human studies, Wahlqvist *et al*.^21^ reported no change in plasma thromboxane B_2_ and platelet aggregation stimulated by ADP and collagen after tocotrienol supplementation. In later studies, Tomeo *et al*.^22^ and Mensink *et al*.^23^ also failed to show that administration of tocotrienols could reduce collagen-induced platelet aggregation.

On the other hand, the saturated family of vitamin E, tocopherols, were reported to exhibit antithrombotic effect in several human trials. Oral administration of α-tocopherol at doses of 400 IU to 1200 IU resulted in attenuation of platelet aggregation via agonist dependent pattern^24, 25^. *In vitro* studies on tocopherols suggested modulation of pathways involving their antioxidant properties^26^, aminophospholipid translocase activity^27^ and protein kinase C-dependent mechanism^28^. Interestingly, tocopherol supplementation was also found to decrease ADP-induced platelet aggregation in type 1 diabetes mellitus patients^29, 30^.

Collectively, the discrepancy in the efficacy of PTT mixture in modulating platelet aggregation is likely to be affected by several factors. First being the dose of tocotrienols. In previous studies, the dose of tocotrienols supplemented ranged from 40 mg to 300 mg per day. Besides, covariate from the studied population and background dietary pattern might affect the study end point. Most importantly, measurement of platelet reactivity was not standardised across the studies. Light transmission aggregometry (LTA) and whole blood aggregometry were found to be the most common methods used. Though being a gold standard method for measurement of platelet aggregation, LTA has been reported with low reproducibility even when the assays were conducted in the same laboratory^31^. Further, platelet-rich plasma samples are needed for measurements using LTA. Several notable disadvantages of using platelet-rich plasma are 1) loss of hyperactive, hypoactive, or giant thrombocytes^32^, injury to platelets or platelets artefact activation could occur during platelet-rich plasma preparation^33^ and 2) measurement is in an artificial milieu lacking leukocytes and erythrocytes under relatively low shear conditions^33, 34^, which influence the results of platelet aggregation. While whole blood aggregometry is designed to circumvent the disadvantages of using platelet-rich plasma, it has been shown to give higher variability than LTA^34^. On the other hand, the concentration of stimulants used in the assays varies among research laboratories and was reported to affect data interpretation significantly. In view of these limitations that may contribute to inconsistent findings, attempt to use an improved instrument is desired for this study. VerifyNow instrument is a point of care instrument that has been designed to overcome the limitations of LTA ﻿utilising whole blood for analysis. It is a cartridge-based system able to minimise concentration and operator variability. Results interpretation using VerifyNow were reproducible and correlated reasonably well with LTA and other types of assays^34–36^. Hence, current study was designed to ascertain the effect of PTT mixture on platelet aggregation in metabolic syndrome subjects, specifically using VerifyNow instrument.

## Materials and Methods

This single centre human trial was conducted in Malaysian Palm Oil Board, Malaysia. Ethical approval was obtained from Medical Research Ethics Committee of Universiti Putra Malaysia, identification number UPM/FPSK/100-9/2-MJKEtikaPen(FBSB_Nov(11)16). The study protocol was registered in ClinicalTrials.gov (NCT01631838) on 26 June 2012. The trial protocol was in compliance with Declaration of Helsinki and Malaysian Guidelines for Good Clinical Practice. Written informed consent was obtained from all participants prior to initiation of screening and trial procedures.

### Participants

The participants were aged between 25 and 60 years old. Metabolic syndrome was defined according to the Clinical Practice Guidelines, Management of Type 2 Diabetes in Malaysia 2009^37^. Participants were identified with waist circumference ≥90 cm in men and ≥80 cm in women and with any two of the following criteria: elevated triacylglycerol (>1.7 mmol/L), low high density lipoprotein (HDL) cholesterol (<1.0 mmol/L in men and 1.3 mmol/L in women), elevated blood pressure (≥130 mm Hg/≥85 mm Hg) or elevated fasting glucose (≥5.6 mmol/L to 7 mmol/L).

The exclusion criteria were as follows: 1) medical history of myocardial infarction, angina, ischemic attack, haemorrhagic stroke, deep vein thrombosis, coronary artery disease, bleeding disorder, or cancer, 2) significant hepatic or renal impairment, 3) fever, cold or infection during bleeding day, 4) fasting serum ferritin below 15 µg/L, 5) fasting haemoglobin below 11.5 g/dL in women and 12.5 g/dL in men, 6) smoker, 7) lactose intolerance, 8) pregnant or breast feeding, 9) alcohol drinker. Subjects were excluded if they were taking vitamin E supplements or medications for anticoagulant, antiplatelet, antihypertensive, glucose lowering, lipid lowering or corticosteroids.

### Trial design

**Figure 1:**
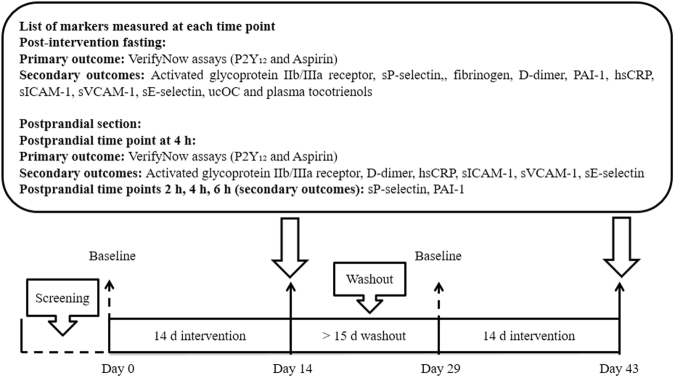
Study design. Abbreviation: hsCRP: high sensitivity C-reactive protein, PAI-1: plasminogen activator inhibitor type 1, sE-selectin: soluble E-selectin, sICAM-1: soluble intracellular adhesion molecules 1, sP-selectin: soluble P-selectin, sVCAM-1: soluble vascular cellular adhesion molecules 1, ucOC: undercarboxylated osteocalcin.

This was a randomised, double-blind, crossover and placebo-controlled trial consisting of two interventions, namely PTT mixture and placebo. Participants were randomised to start with any one of the two interventions using Latin square design generated in computer. Participants and investigators were blinded from the allocation of interventions. Each intervention involved a supplementation period of 14 days separated by a washout period of at least 15 days (Fig. 1). During PTT mixture intervention, participants consumed one capsule of 200 mg Tocovid^TM^ *SupraBio* ^TM^ (containing 61.52 mg α-tocotrienol, 112.80 mg γ-tocotrienol, 25.68 mg δ-tocotrienol and 61.07 mg α-tocopherol, batch no: 11901BBA) twice daily after breakfast and dinner. Similarly, during placebo intervention, participants consumed one placebo capsule (palm olein containing <1 mg vitamin E, batch no: 11902BBA) twice daily. Both types of capsules were similar in physical appearance and were supplied in colour coded bottles (Hovid Bhd., Malaysia). As vitamin E absorption could vary with the amount of fat intake, participants were instructed to consume 125 mL of full cream milk containing 4.3 g of fat together with the capsules. One day before study visit, participants were instructed to avoid strenuous exercise, consumption of alcohol, caffeine, and high fat dinner, and to fast after 10.00 pm.

Blood samples were collected on day 0 (baseline fasting blood sample) and day 14 (post-intervention fasting blood sample). On postprandial day, participants were given a high fat breakfast consisting of one high fat muffin and a glass of 100 mL milkshake (providing approximately 828 kcal energy, 54 g fat, 73 g carbohydrate, and 12 g protein) before consuming one test capsule. Blood samples were collected at 2 h, 4 h, and 6 h (Fig. 1). Participants were allowed to sip water (<600 mL) but not allowed to eat over the postprandial period. Lunch was given to participants after blood sampling. Participants were advised to maintain their usual lifestyle and diet throughout the study period. They were requested to record any illness, medication used and study deviation during study period, and notify investigator at each study visit. Any usage of medications that could interfere with the results of this study was not allowed. If the medication were critical for participant’s health, subjects were dropped out.

### Compliance measurement

Measurement of compliance was conducted via pill counting of returned bottles from the participants during post-intervention visit. Plasma tocotrienol levels were also measured using high performance liquid chromatography method as previously described by Che *et al*.^38^.

### Primary outcome - VerifyNow assays

VerifyNow instrument (Accumetrics, Inc., California, USA) measures platelet aggregation reactivity based on the ability of activated platelets binding towards fibrinogen-coated beads in the presence of specific agonist. This rapid automated cartridge-based analyser was attached either with VerifyNow Aspirin cartridge or VerifyNow P2Y_12_ cartridge during assays^39^. As for the VerifyNow Aspirin cartridge, platelet aggregation in whole blood sample was induced by arachidonic acid, and the results were expressed as aspirin reactivity unit (ARU). Whereas in VerifyNow P2Y_12_ cartridge, ADP was used to stimulate platelet aggregation, and the results were expressed as P2Y_12_ reactivity unit (PRU). During analysis, whole blood samples were collected into a 2 mL 3.2% sodium citrate tubes and inverted five times. Subsequently, the samples were incubated at room temperature for 10 min and 30 min for VerifyNow P2Y_12_ and VerifyNow Aspirin assays, respectively.

### Secondary outcomes – Blood, serum and plasma assays

Activated glycoprotein IIb/IIIa receptor, which is expressed only on the activated platelet surface, was determined using a method derived from Furman *et al*.^40^. Whole blood sample was collected into a tube contained 3.2% sodium citrate and processed within 30 min. A volume of 5 µL of anticoagulated whole blood was added into a polypropylene tubes containing 20 µM (DL-Isoser1)-thrombin receptor activating peptide-6 trifluoroacetate salt (thrombin mimic peptide, Bachem AG, Switzerland), fluorescein isothiocyanate conjugated PAC-1 (PAC-1-FITC) (Becton, Dickinson and Company, New Jersey, USA) and peridinin chlorophyll protein complex conjugated CD61 (CD61-PerCP) (Becton, Dickinson and Company, New Jersey, USA). Sample was gently swirled for mixing and incubated at room temperature for 20 min. The mixture was then fixed with 0.5% formalin at a pH of 7.4 (10 mmol/L HEPES buffer, 0.15 mM sodium chloride). The fixed sample was placed at 4 °C for at least 30 min before analysing with a BD FACSCalibur™ flow cytometer (Becton, Dickinson and Company, New Jersey, USA). Platelets were identified by CD61-PerCP, and data were obtained for 10,000 platelet events. The activated glycoprotein IIb/IIIa event was determined based on the mean fluorescence intensity (MFI) of PAC-1-FITC antibody binding on the dual parameter dot plot of PAC-1-FITC fluorescence displaying events from CD61 positive region.

Plasma plasminogen activator inhibitor type 1 (PAI-1) and D-dimer were determined using IMUBIND® plasma PAI-1 ELISA kit (Sekisui Diagnostics, LLC., USA) and IMUCLONE® D-dimer ELISA kit (Sekisui Diagnostics, LLC., USA), respectively. Plasma soluble P-selectin (sP-selectin), soluble E-selectin (sE-selectin), soluble intracellular adhesion molecule 1 (sICAM-1) and soluble vascular cellular adhesion molecule 1 (sVCAM-1) were analysed using Human sP-selectin/CD62P Immunoassay kit (R&D Systems, Inc., USA), Quantikine® Human sE-selectin/CD62E Immunoassay kit (R&D Systems, Inc., USA), Quantikine® Human sICAM-1/CD54 Immunoassay kit (R&D Systems, Inc., USA) and Quantikine® Human sVCAM-1 Immunoassay kit (R&D Systems, Inc., USA), respectively. Plasma undercarboxylated osteocalcin (ucOC) was measured using ucOC EIA kit (Takara Bio Inc., Japan). Assay of plasma fibrinogen was carried out using Sysmex automated haematology analyser XT-4000*i* (Sysmex Corporation, Kobe, Japan). Serum high sensitivity C-reactive protein (hsCRP) assay was performed using ADVIA® 2400 Clinical Chemistry System autoanalyser (Siemens AG, Munich, Germany).

### Sample size calculation and statistical analysis

Sample size was calculated based on 95% power at P = 0.01 to detect a 8% of mean reduction in ADP stimulated platelet aggregation (estimated from Qureshi *et al*.^16^) based on fasting platelet aggregation units (PAU) in metabolic syndrome reported by Serebruany *et al*.^1^. All data were presented as means ± standard deviations (SDs). The normality of data distribution was examined using D’Agostino & Pearson omnibus test in GraphPad Prism software (Version5.01; GraphPad Software, Inc., California, USA). Data was assumed normally distributed when P > 0.05 based on 95% confidence interval. Logarithmic transformation was performed for several parameters as stated in the Results section, with data shown being original values. Differences between means were tested with Student’s paired t-test for data distributed normally. Non-parametric test (Wilcoxon Signed Ranks test) was used for the statistical analysis of data that were different from Gaussian distribution. Repeated measures generalised linear model with Bonferroni test was performed for postprandial PAI-1 and sP-selectin. All data was assumed significantly different at P < 0.05 based on the confidence interval of 95%. These statistical analyses were performed using IBM SPSS statistical software (version 20; SPSS, Inc., Illinois, USA).

## Results

**Figure 2:**
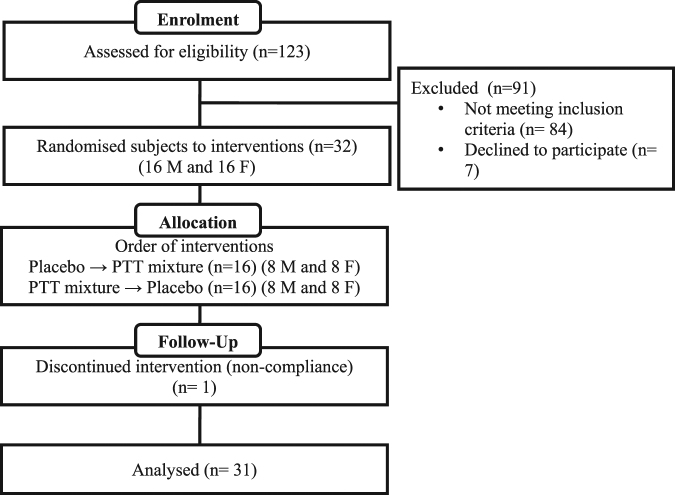
Consort diagram.

Figure 2 showed the consort diagram of this study. Recruitment for the study were carried out between early April 2012 and mid of July 2012. Out of 123 individuals who attended the screening, 32 metabolic syndrome subjects who met the participation criteria were recruited in this crossover study. One male volunteer had discontinued from the study due to non-compliance. Table 1 summarised the baseline characteristics of 31 subjects who completed the study and on whom data were available for the statistical analysis of primary outcomes.

**Table 1:** Baseline characteristics of study population.

	Men (n = 15)	Women (n = 16)
Ethnicity		
Malay	9 (60.0%)	14 (87.5%)
Chinese	4 (26.7%)	0 (0.0%)
Indian	2 (13.3%)	2 (12.5%)
Age (y)	34 ± 8.2	41.6 ± 10.7
Weight (kg)	86.4 ± 14.6	73.4 ± 15.3
BMI (kg/m^2^)	29.4 ± 5.3	30.5 ± 5.4
Waist circumference (cm)	100.9 ± 9.4	96.5 ± 7.7
Blood pressure (mm Hg)		
SBP	133.4 ± 7.8	133.2 ± 15.2
DBP	86.4 ± 7.6	85.2 ± 9.5
Serum triacylglycerol (mmol/L)	2.1 ± 0.7	1.6 ± 0.7
Serum HDL cholesterol (mmol/L)	1.0 ± 0.1	1.2 ± 0.1
Fasting glucose (mmol/L)	5.1 ± 0.5	5.1 ± 0.4

Abbreviation: BMI: body mass index, DBP: diastolic blood pressure, SBP: systolic blood pressure, HDL: high density lipoprotein.

No serious adverse events were reported throughout the study period. Subjects’ body weights were relatively stable recording approximately 0.01% changes. Compliance via pill counting showed an average of 99.5% (1.99 capsules/d) and 98.4% (1.97 capsules/d) compliance for PTT mixture and placebo interventions, respectively. Fasting plasma tocotrienol concentration in PTT mixture group (0.58 ± 0.50 µg/mL) was found to be significantly higher (p < 0.001) than placebo group (0.03 ± 0.06 µg/mL).

### Primary outcome

**Figure 3:**
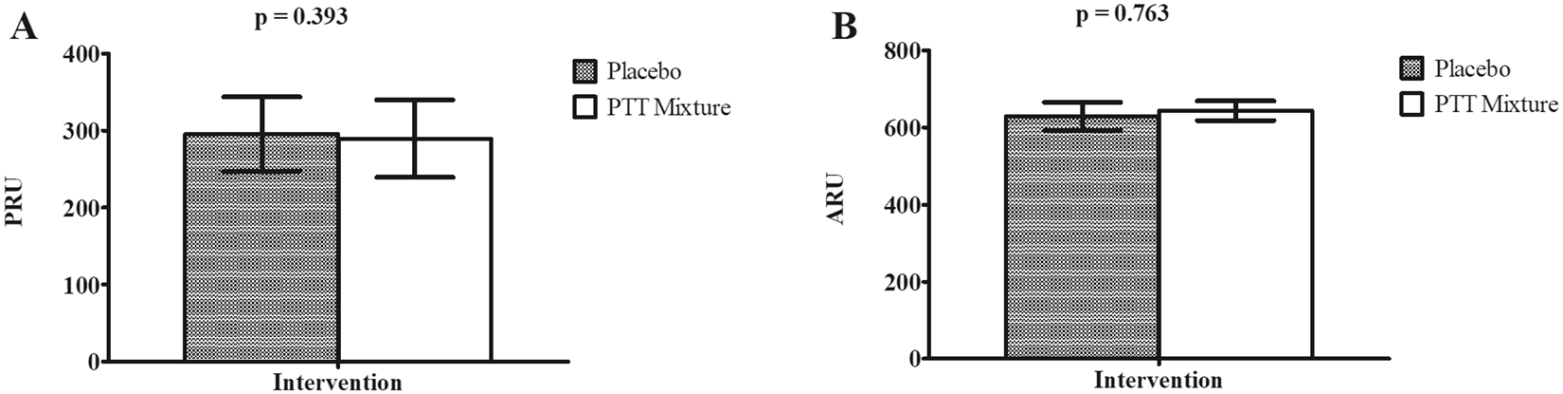
Post-intervention platelet aggregation reactivity results of PRU (**A**) and ARU (**B**). (n = 31; 15 males and 16 females). Intervention effect of PRU and ARU was examined using Student t-test. Abbreviation: ARU: Aspirin reactivity units, PRU: P2Y_12_ reactivity units.

Following 14 days of supplementation, results of VerifyNow P2Y_12_ assay demonstrated no significant difference in ADP-induced platelet aggregation reactivity between PTT mixture and placebo interventions (290 ± 50 PRU vs 295 ± 48 PRU, p = 0.393) (Fig. 3 Panel A). Arachidonic acid induced platelet aggregation reactivity, as measured by VerifyNow Aspirin assay, were 631 ± 33 ARU and 628 ± 36 ARU in PTT mixture and placebo interventions respectively, with no significant difference among interventions (p = 0.763) (Fig. 3 Panel B).

**Figure 4:**
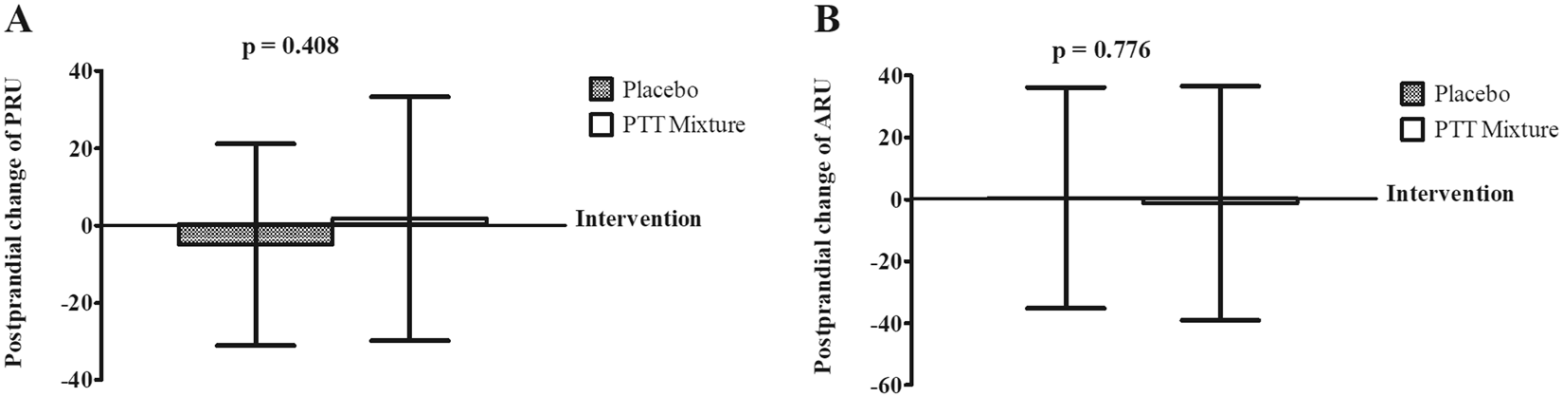
Postprandial change of platelet aggregation reactivity of PRU (**A**) and ARU (**B**). (n = 31; 15 males and 16 females). Intervention effect of PRU and ARU was examined using Student t-test and Wilcoxon Signed Rank test, respectively. Abbreviation: ARU: Aspirin reactivity units, PRU: P2Y_12_ reactivity units.

A postprandial study was carried out to examine the acute response of platelet aggregation reactivity following PTT mixture supplementation (Fig. 4). Changes in postprandial platelet aggregation induced by ADP were found to be 1.77 ± 31.59 PRU and −4.97 ± 26.11 PRU in PTT mixture and placebo interventions, respectively (Fig. 4 Panel A). As for platelet aggregation induced by arachidonic acid, postprandial changes did not differ significantly in PTT mixture and placebo interventions, i.e. −1.29 ± 37.77 ARU vs 0.45 ± 35.63 ARU, p = 0.776 (Fig. 4 Panel B).

### Secondary outcome

Post-intervention results for secondary outcome were summarised in Table 2. Measurements of glycoprotein IIb/IIIa receptor activation using flow cytometry method did not show significant difference between PTT mixture and placebo groups (12.37 ± 3.42 MFI vs 12.09 ± 3.53 MFI, p = 0.602). Plasma sP-selectin, a marker representing *in vivo* platelet activation status, was measured at 93.96 ± 67.53 ng/mL and 78.60 ± 43.97 ng/mL for PTT mixture and placebo interventions, respectively. A slight decrease in fasting D-dimer levels was noted in the PTT mixture group compared to placebo, although the difference did not reach statistical significance (p = 0.505). Additional thrombogenic markers including fibrinogen, PAI-1 and ucOC were found to be similar between the two intervention groups. Measurements of soluble inflammatory (hsCRP) and adhesion molecules (sE-selectin, sICAM-1 and sVCAM-1) did not show notable changes throughout the interventions.

**Table 2:** Effect of PTT mixture and placebo supplementations for 14 days on platelet activation, haemostatic and inflammatory markers.

	PTT Mixture Intervention	Placebo Intervention	P
Activated glycoprotein IIb/IIIa receptor (MFI)	12.37 ± 3.42	12.09 ± 3.53	0.602
sP-selectin (ng/mL)	93.96 ± 67.53	78.60 ± 43.97	0.224
Fibrinogen (g/L)	3.00 ± 0.68	2.97 ± 0.69	0.865
D-dimer (ng/mL)	241.82 ± 170.72	287.80 ± 418.08	0.505
PAI-1 (ng/mL)	78.69 ± 33.91	80.11 ± 33.30	0.551
ucOC (ng/mL)	4.74 ± 2.10	4.90 ± 1.98	0.196
hsCRP (mg/L)	4.81 ± 4.86	4.94 ± 4.65	0.795
sE-selectin (ng/mL)	43.55 ± 15.02	43.45 ± 15.99	0.715
sICAM-1 (ng/mL)	220.20 ± 56.62	219.20 ± 63.68	0.629
sVCAM-1 (ng/mL)	554.60 ± 138.00	576.00 ± 149.00	0.210

Prior to statistical analysis, data for activated glycoprotein IIb/IIIa receptor, fibrinogen, sE-selectin, sICAM-1 and hsCRP were logarithmic transformed. Data for activated glycoprotein IIb/IIIa receptor, fibrinogen, PAI-1, sE-selectin, sICAM-1 and hsCRP were analysed using Student t-test while data for D-dimer, ucOC, sP-selectin and sVCAM-1 were analysed using Wilcoxon Signed rank test. Abbreviation: hsCRP: high sensitivity C-reactive protein, PAI-1: plasminogen activator inhibitor type 1, sE-selectin: soluble E-selectin, sICAM-1: soluble intracellular adhesion molecules 1, sP-selectin: soluble P-selectin, sVCAM-1: soluble vascular cellular adhesion molecules 1, ucOC: undercarboxylated osteocalcin.

**Figure 5:**
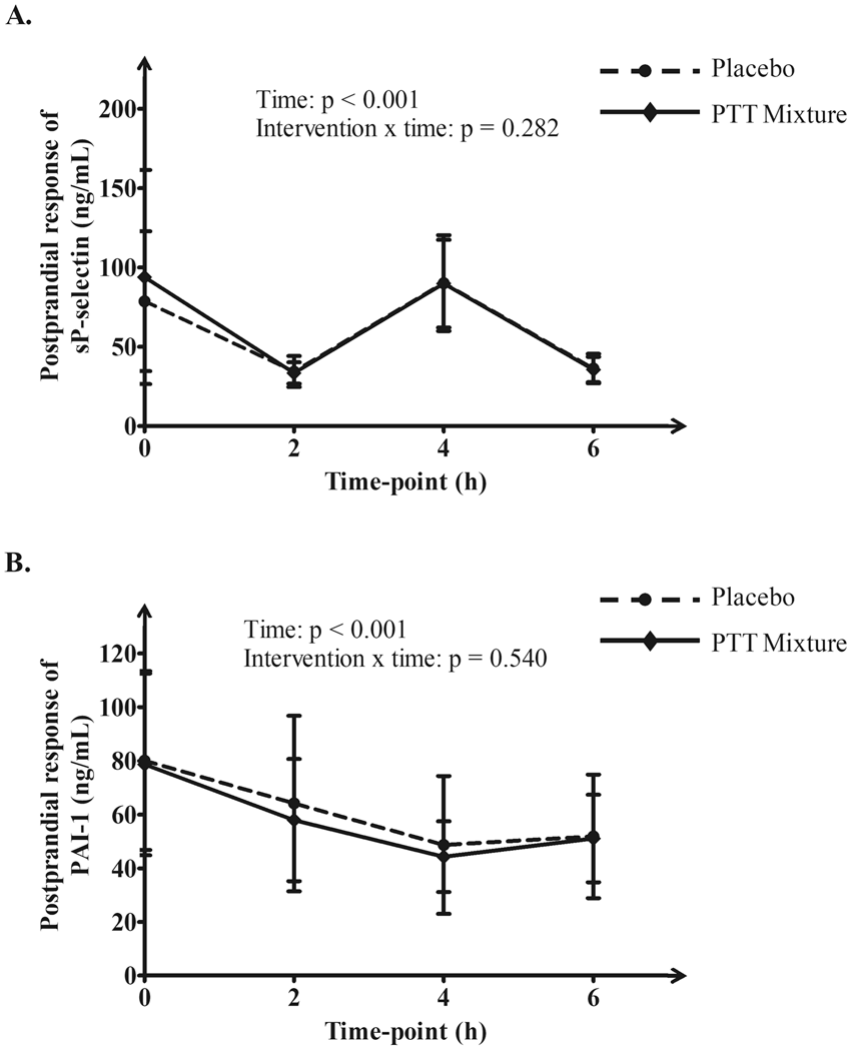
Postprandial response of plasma sP-selectin (**A**) and plasma PAI-1 (**B**). Only data for PAI-1 was logarithmic transformed during statistical analysis. The intervention and time effects were analysed using repeated measures generalised linear model. Abbreviation: PAI-1: plasminogen activator inhibitor type 1, sP-selectin: soluble P-selectin.

Table 3 summarised the postprandial changes of secondary outcome. Minimal changes were observed in postprandial samples of glycoprotein IIb/IIIa receptor activation, D-dimer, hsCRP, sE-selectin and sVCAM-1 (p > 0.05). Plasma sICAM-1 concentration was significantly lowered in PTT mixture group compared to placebo group (−5.27 ± 15.28 ng/ml vs 2.66 ± 14.56, p = 0.046) during postprandial measurements. Figure 5 illustrated the postprandial responses of sP-selectin and PAI-1 after a high fat meal and test capsules. A significant time effect (p < 0.001) was observed for sP-selectin for both PTT mixture and placebo interventions. A drop in sP-selectin levels (64.6% and 56.5% from fasting levels in PTT mixture and placebo groups) was recorded at 2 h postprandial, followed by an increase at 4 h. However, no significant difference was observed when analysed for intervention × time effect (p = 0.282) (Fig. 5 Panel A). As illustrated in Fig. 5 Panel B, postprandial plasma PAI-1 decreased by 43.6% and 39.3% from fasting levels for PTT mixture and placebo interventions at 4 hr. Similar to the trend of sP-selectin, PAI-1 levels recorded a slight increase at 6 h postprandial. Thus, a significant difference was detected for time effect (P < 0.001). When analysed for intervention × time effect, no statistical significance was observed (p = 0.540).

**Table 3:** Postprandial change (4 hour) of platelet function values, haemostatic and inflammatory markers.

	PTT Mixture Intervention	Placebo Intervention	P
Activated glycoprotein IIb/IIIa receptor(MFI)	−1.59 ± 2.89	−0.70 ± 2.92	0.298
D-dimer (ng/mL)	30.72 ± 73.42	15.08 ± 109.4	0.814
hsCRP (mg/L)	0.11 ± 0.68	0.21 ± 1.28	0.871
sE-selectin (ng/mL)	−1.49 ± 2.36	−0.79 ± 2.90	0.286
sICAM-1 (ng/mL)	−5.27 ± 15.28*	2.66 ± 14.56*	0.046
sVCAM-1 (ng/mL)	−19.92 ± 33.29	−19.42 ± 43.11	0.952

Interventions effect of activated glycoprotein IIb/IIIa receptor and sVCAM-1 were analysed using Student t-test. While intervention effect of D-dimer, hsCRP, sE-selectin and sICAM-1 were analysed using Wilcoxon Signed Rank test. Abbreviation: hsCRP: high sensitivity C-reactive protein, sE-selectin: soluble E-selectin, sICAM-1: soluble intracellular adhesion molecules 1, sVCAM-1: soluble vascular cellular adhesion molecules.*Indicated p < 0.05 between interventions.

## Discussion

ADP is a platelet agonist released from dense granules of activated platelets. It activates platelets through two purinegic receptors, which are P2Y_12_ and P2Y_1_^41^. Several studies investigated the effects of tocotrienol supplementation on ADP-induced platelet aggregation, but these results are inconsistent. Qureshi *et al*.^16^ demonstrated significantly lower percentage of platelet aggregation in response to ADP after 4 weeks supplementation of 200 mg tocotrienol-rich fraction in hypercholesterolemic subjects. Pronounced inhibition effect on ADP induced platelet aggregation was also observed in both α-tocotrienol and tocotrienol-rich fraction treatments in their animal study using canine model^15^. On the contrary, in a human study conducted by Wahlqvist *et al*.^21^ where subjects were supplemented for 16 weeks with increasing doses of tocotrienol-rich fraction (60 to 240 mg/day) no significant reduction in platelets aggregability responded to ADP activation was reported. The variability results between these studies may be due to variation of methods and different concentrations of ADP used in platelet aggregation measurement using LTA. While Wahlqvist *et al*.^21^ investigated platelet aggregation using ADP concentrations ranging from 10 to 62.5 μM, Qureshi *et al*.^16^ used 5 and 20 μM of ADP as stimulants, correlating to up to 3-fold difference between the two studies. Unlike previous studies, VerifyNow instrument, a fully automated cartridge-based instrument that measures platelet aggregation at a standardised condition with its cartridge-based design and at a fixed concentration of ADP was used in current trial. In the VerifyNow P2Y_12_ cartridge, platelets are activated by ADP (20 μM) in the presence of prostaglandin E_1_ (22 nM)^42^. Prostaglandin E_1_ is an adenylyl cyclase stimulator, which inhibits calcium ion releases, thus suppresses the platelet activation induced via P2Y_1_ receptor to enhance specificity and sensitivity of ADP activation through P2Y_12_ receptor^43^. Results of current study suggested that tocotrienols did not modulate platelet aggregation through P2Y_12_ receptor following 14 days of PTT mixture supplementation in metabolic syndrome population. Similar observations were found during fasting and postprandial states.

Platelet aggregation can also be mediated through arachidonic acid signalling pathway. During platelet activation, arachidonic acid is released from phospholipids and converted to thromboxane A_2_ through a series of enzyme activities. Thromboxane A_2_ will then bind to thromboxane receptor to activate platelets^44^. In order to measure the effect from this pathway, a surrogate marker, thromboxane B_2_ was commonly determined being a stable metabolite derived from thromboxane A_2_. In two human studies, plasma and urine thromboxane B_2_ levels did not show significant changes after tocotrienol supplementation^21, 22^. In line with the results from these studies, PTT mixture supplementation for 14 days and postprandially did not exert any inhibitory effect on arachidonic acid induced platelet aggregation using VerifyNow system. However, marked reduction of serum or plasma thromboxane B_2_ after tocotrienol supplementation was found in studies conducted by Qureshi *et al*.^17, 18^. It is notable that diet control (American Heart Association Step 1 diet or National Cholesterol Education Program Step 1 diet) was implemented in the studies of Qureshi *et al*.^17, 18^, limiting the intake of fat, cholesterol and total calorie. It was postulated that a combinatorial effect on the inhibition of platelet aggregation might be observed with PTT mixture and diet control.

For secondary outcomes, platelet activation was monitored via thrombin mimic peptide agonist and plasma sP-selectin levels. Thrombin is a platelet agonist generated in the coagulation system which binds to protease-activated receptor-1 and protease-activated receptor-4 on platelet’s surface, subsequently triggering the cascade events of platelet aggregation^44^. An *in vitro* study with tocopherol showed a dose dependent involvement of aminophospholipid translocase activity induced by thrombin, which was responsible for the inhibitory effect in platelet aggregation^27^. In this study, thrombin-induced activation of glycoprotein IIb/IIIa receptors was measured using flow cytometry. Our results indicated no changes in platelet activation in whole blood samples from subjects taking PTT mixture. The results were correlated to a study conducted by Tomeo *et al*.^22^ where subjects with hyperlipidemia and carotid stenosis consumed tocotrienols in an increasing dose manner (224 mg/day – 336 mg/day) for 18 months. Measurements of platelet activation via platelet adenosine triphosphate release induced by thrombin did not show significant reduction. In the same study, changes in platelet aggregation induced by collagen were not significant in groups taking palm mixed tocotrienols and tocopherol compared to placebo. Similar data on collagen-induced platelet aggregation was reported in Mensink *et al*.^23^. Future study on collagen-induced platelet aggregation after tocotrienol supplementation is warranted to confirm its effect. As for sP-selectin, plasma levels were measured at fasting and postprandial states, being an indicator of *in vivo* platelet activation status. Although time dependent changes at 6-hour postprandial was observed, PTT mixture supplementation for 14 days did not modulate sP-selectin levels compared to placebo group. As for haemostatic markers, results obtained were in consistent with platelet aggregation and platelet activation. Biomarkers measured in this study include D-dimer, PAI-1 and fibrinogen. D-dimer is a biomarker that reflects activation of coagulation and fibrinolysis^45^, while PAI-1 is a regulator of D-dimer concentration, which inhibits the initiation of fibrinolysis process^46^. Fibrinogen is a precursor for fibrin formation and glycoprotein that bridging the activated platelets via activated glycoprotein IIb/IIIa receptors to promote platelet aggregation^44^. These findings were in accordance with results demonstrated by Mensink *et al*.^23^, suggesting the lack of influence from tocotrienols on the coagulation system. In Mensink *et al*.^23^, no changes were found in D-dimer, PAI-1, Factor VII, fibrinogen, fragment 1 + 2 and antithrombin III. On the other hand, plasma ucOC is a sensitive measure of vitamin K status^47^, in which vitamin K acts as an important cofactor in the coagulation cascade. Our results showed minimal changes in ucOC levels in both groups, indicating the lack of interaction between tocotrienols and the vitamin K cascade in affecting the coagulation status. As exploratory outcome, this study measured several plasma inflammatory markers at fasting and 4-hour postprandial. The results did not show improvement on inflammation status based on plasma concentrations of inflammatory markers, i.e. sE-selectin, sVCAM-1 and hsCRP. A slight reduction was observed in 4-hour postprandial levels of sICAM-1 in the PTT mixture group, although the difference was minimal with relatively large SD. However, soluble fractions of these biomarkers in the plasma were measured using ELISA method. Our results leave room for doubt on their expression profile on platelets especially for indicators of platelet function including P-selectin and ICAM-1. Future study using flow cytometry method is warranted.

From another point of view, there are several potential limitations in this study. First being the duration of study with supplementation period of 14 days. The supplementation period was proposed to be 14 days in this study in view that: i) physiological life span of blood platelets is about 7 to 10 days with a daily renewal rate of about 20% of total platelet count^48^, ii) maximal inhibition of platelet aggregation following oral administration of platelet aggregation inhibition agents occurred within 5 days^49^ and iii) concentration of tocotrienols in platelet was able to double after 10 days of tocotrienol (80 mg/d) supplementation^50^. However, taken from previous studies that tocotrienols were able to improve lipid profiles after 6 months supplementation^51^, the supplementation period of 14 days in this study might be relatively too short for tocotrienols to exhibit platelet-aggregating inhibitory effect. Further, the effect of tocotrienols in protection against white matter lesion was only noted after 2 years supplementation of palm-mixed tocotrienols^52^. Although not investigated in this study, measurement of tocotrienols incorporation into platelet fractions would have provided interesting perspective complementing measurement of tocotrienol levels in plasma. *In vitro*, Freedman *et al*.^28^ reported a dose and time dependent increment of α-tocopherol from 0.25 to 2.5 mM and 2 to 30 minutes, respectively. In these experiments, high levels of platelet α-tocopherol were correlated with lower extent of platelet aggregation. In a separate study, Hayes *et al*.^50^ conducted a short term supplementation study with palm-mixed tocotrienols and tocopherol. Similar trend was observed in plasma and platelet levels of tocotrienols after 10 days supplementation (80 mg tocotrienols and 64 mg α-tocopherol). The percentage of increment from baseline was higher in tocotrienol levels (272% in plasma and 96% in platelet) compared to tocopherol levels (69% in plasma and 35% in platelet). Secondly, it was unclear if the presence of tocopherol at 31% had a possible impact on the transport mechanism of tocotrienols. It was reported by Shibata *et al*.^53^ in a rat feeding trial that α-tocopherol at 50 mg/day attenuated the cholesterol-lowering effect of rice bran tocotrienols at 11 mg/day. The effect corroborated with a decrease in peripheral tissue concentrations of tocotrienols after coadministration with tocopherols, suggesting possible inhibitory mechanism on the absorption pathways. Nevertheless, the dose of α-tocopherol administered in this study (122 mg, equivalent to 91 IU per day) was lower than published results reporting at 400 to 1200 IU. Besides, several studies reported the lack of inhibitory effect on platelet aggregation with α-tocopherol compared to mixed tocopherols that contained high concentration of γ-tocopherol^54, 55^. In summary, this study implied that acute supplementation of PTT mixture at a dose of 400 mg/day did not affect the modulation of platelet aggregation, platelet activation, coagulation and inflammatory status in subjects with metabolic syndrome. All subjects well-tolerated the 2 weeks PTT mixture supplementation without any adverse events being reported. Our results implied the lack of clinically significant effect on platelet homeostasis with acute supplementation of PTT mixture. Information from this study would add value to health practitioners and this cohort intending to consume PTT mixture as supplements for a short period of time. Nevertheless, their long term effects on platelet function remained uncertain and should be warranted in future studies.
